# B2M or CIITA knockdown decreased the alloimmune response of dental pulp stem cells: an in vitro study

**DOI:** 10.1186/s13287-024-04023-5

**Published:** 2024-11-14

**Authors:** Mingxin Hu, Yuchen Zhang, Junqing Liu, Yihan Chen, Jun Kang, Jialin Zhong, Shulan Lin, Ye Liang, Rong Cen, Xiaofei Zhu, Chengfei Zhang

**Affiliations:** 1 Restorative Dental Sciences, Endodontics, Faculty of Dentistry, The University of Hong Kong, Hong Kong, SAR China; 2 Obstetrics Department, The Third Affiliated Hospital of Zhengzhou University, Zhengzhou, China; 3 Department of Endodontics, Henry M. Goldman School of Dental Medicine, Boston University, Boston, USA

**Keywords:** Dental pulp stem cells, Beta 2-microglobulin, Class II histocompatibility complex transactivator, Human leukocyte antigen, Immune rejection

## Abstract

**Background:**

Dental pulp stem cells (DPSCs) have acquired noteworthy attention for their application in treating ischemic diseases and facilitating tissue regeneration. However, the host’s immune response following allogenic DPSC transplantation often handicaps the long-term survival of transplanted cells, thereby limiting the application of DPSCs in cell therapy. This study aims to investigate whether genetic modification can alleviate the immunogenicity of DPSCs.

**Methods:**

Beta 2-microglobulin (B2M) and the class II histocompatibility complex transactivator (CIITA) were individually knocked down in DPSCs by lentiviral particles encoding short hairpin (sh) RNAs. The self-renewal capacity and pluripotency of DPSCs-shB2M (B2M silenced DPSCs) and DPSCs-shCIITA (CIITA silenced DPSCs) were evaluated by CCK8 and differentiation assays including osteogenesis, adipogenesis, and neurogenesis. The expression of HLA-I and HLA-II in DPSCs-shB2M and DPSCs-shCIITA after IFN-γ treatment were analyzed by western blotting, immunofluorescence, and flow cytometry. The function of genetically modified cells was assessed by leukocyte-mediated cytotoxicity and T-cell proliferation assays.

**Results:**

Western blotting, immunofluorescence, and flow cytometry revealed that DPSCs-shB2M and DPSCs-shCIITA exhibited impaired IFN-γ inducible HLA-I and HLA-II expression. There were no significant differences in the self-renewal capacity and pluripotency among DPSCs-shB2M, DPSCs-shCIITA, and control groups (*p* > 0.05). Lower leukocyte-mediated cytotoxicity and higher cell survival rates were found in DPSCs-shB2M and DPSCs-shCIITA groups compared to the control (*p* < 0.05). T cell proliferation was significantly inhibited in both DPSCs-shB2M and DPSCs-shCIITA groups (*p* < 0.05).

**Conclusion:**

Genetic knockdown of B2M or CIITA in DPSCs substantially reduced their immunogenicity without compromising their stemness, thereby broadening the clinical application of DPSCs in cell therapy and tissue regeneration.

**Supplementary Information:**

The online version contains supplementary material available at 10.1186/s13287-024-04023-5.

## Introduction

Dental pulp stem cells (DPSCs) are a promising cell type for oral tissue regeneration, including pulp, periodontium, and alveolar bone [1–4]. Additionally, their therapeutic potential extends to treating conditions such as cardiac infarction and cerebral ischemic injury [5, 6]. Derived from mesenchymal stem cells (MSCs), DPSCs share critical characteristics with them, such as self-renewal ability and multilineage differentiation capacity [7]. DPSCs are also considered immunoprivileged due to their negligible expression or absence of major histocompatibility complex (MHC) class II molecules [8, 9]. The immunoprivilege enables allogeneic DPSC transplantation without the risk of immune rejection [10]. However, studies have revealed challenges in the long-term survival of transplanted DPSCs in animal models [11, 12]. Intramyocardially injected bone marrow-derived MSCs showed only 6% retention after ten days in a porcine ischemic myocardium model [13]. Similarly, human DPSCs (hDPSCs) transplanted into the ischemic region of the post-stroke Sprague–Dawley rats model only achieved 2.3% survivability at 4 weeks [14]. Although there are various factors affecting transplanted cell survivability, such as graft design and engraftment efficiency, these studies suggest that one of the possible reasons for the low retention rate of transplanted MSC is their immunogenicity in stressful microenvironments or upon cell differentiation, thus rendering them susceptible to host immune system rejection despite their initial immune privileged [15, 16]. To maximize the therapeutic potential of allogeneic DPSCs, it is crucial to maintain the immune privilege to enhance their long-term survivability after allotransplantation.

Evidence from in vivo studies has shown that a mismatch in the MHC during MSC transplantation could trigger both cell-mediated and humoral immune responses, resulting in rejection [12, 17]. Additionally, a clinical trial revealed that 34% of patients treated with allogeneic MSCs developed anti-HLA Class I antibodies after 3 months, as compared to the placebo-treated group [18]. These findings collectively indicate that human leukocyte antigens (HLAs), known as the MHC system in humans, are primary mediators of immune rejections. HLAs consist of class I (HLA-I, expressed on the surface of almost all nucleated cells) and class II (HLA-II, expressed on B lymphocytes, antigen-presenting cells, activated T lymphocytes, etc.) molecules. While DPSCs constitutively express HLA-I, they typically lack HLA-II expression. However, the harsh microenvironment of hypoxia or cell differentiation could induce HLA-II expression [19–21]. The ischemic environment also upregulated HLA-I expression [22]. It has been documented that the degree of mismatch in MHC-I and MHC-II antigens between the donor and recipient was positively associated with the intensity of the resulting immune response [23]. The increased expression of both HLA-I and HLA-II triggered an allogeneic T cell-mediated immune response, subsequently disrupting the immune privilege of the transplanted cells and compromising their survival [24, 25]. Therefore, generating a DPSC cell line with low HLA expression could be an alternative way to address immune rejection associated with DPSC allotransplantation.

Beta 2-microglobulin (B2M) is the light chain in HLA-I. The complete inactivation of the B2M gene completely disabled the HLA-I molecule's function [26]. MHC-II transactivator (CIITA) serves as the dominant regulator of HLA-II molecules [25], and without it, HLA-II transcription decreases [27]. Silencing B2M and CIITA in MSCs via genetic modification is expected to reduce HLA-I and HLA-II expression, thereby decreasing the immunogenicity of transplanted cells and significantly increasing their survival. This study aimed to investigate the effects of B2M or CIITA knockdown on the immunogenicity of DPSCs in vitro. The hypothesis was that knocking down of B2M or CIITA would decrease DPSC immunogenicity without affecting their self-renewal capacity and pluripotency in vitro.

## Materials and methods

This study was conducted following the ethical guidelines approved by the Institutional Review Board of the University of Hong Kong/Hospital Authority Hong Kong West Cluster (IRB Ref. Number: UW 22-611) for collecting human peripheral blood mononucleated cells (PBMCs).

### Cell culture

Human DPSCs (hDPSCs) were obtained from Lonza (Basel, Switzerland) and were cultured in α-modified Eagle's medium (α-MEM) (Thermo Fisher Scientific, Waltham, MA, USA) supplemented with 10% (v/v) fetal bovine serum (FBS) (Thermo Fisher Scientific) and 1% (v/v) P/S (penicillin/streptomycin) (Sigma-Aldrich, St. Louis, MO, USA). Cells between passages 3 to 6 were incubated in 5% CO_2_ at 37 °C for further experiments.

### Time-dependent treatments in hDPSCs

A series of time-dependent analyses were conducted on the target protein expression levels in hDPSCs after IFN-γ treatment to optimize the expression of B2M, CIITA, HLA-I, and HLA-II in subsequent experiments. The following were the details of the methodology:

To quantify the total protein expression of B2M, CIITA, HLA-I, and HLA-II in hDPSCs, cells were seeded in the 6-well plate at a density of 3 × 10^5^ cells/well and treated with IFN-γ (10 ng/mL) in α-MEM supplemented with 10% (v/v) fetal bovine serum (FBS) and 1% (v/v) P/S (penicillin/streptomycin) under 5% CO_2_ at 37 °C. Samples were collected for Western blotting analysis after incubation at 0, 2, 4, 6, 8, and 24 h.

To track the localization of HLA-I and HLA-II in hDPSCs, cells were seeded in 12-well plate (1 × 10^5^ cells/well) and cultured in α-MEM/10% FBS with or without IFN-γ (10 ng/mL) for 1, 3, 5, and 7 days; samples were then fixed with 100% iced methanal for immunofluorescence analysis.

To measure the cell surface protein expression of HLA-I and HLA-II in hDPSCs, cells were seeded in a 60-mm culture dish (Corning™, Corning, New York, USA) at a density of 3 × 10^5^ cells/dish. They were treated in the α-MEM/10% FBS with or without IFN-γ (10 ng/mL). The medium was refreshed bi-daily. Samples were collected for flow cytometry analysis after 1, 3, 5, and 7 days.

### Knockdown of B2M or CIITA in hDPSCs

B2M or CIITA knockdown was achieved using shRNA particles and the respective control-vector lentiviral particles (Santa Cruz Biotechnology, Texas, USA). Lentiviral transduction was performed as per the manufacturer's protocol. Briefly, hDPSCs were plated in a 6-well plate with 50% confluence. After attachment, Polybrene (Santa Cruz Biotechnology) at a concentration of 5 μg/mL was used to enhance infection efficiency, followed by adding 20 μL lentiviral particles (1 × 10^5^ IFU) into the culture medium each well. Stably transduced hDPSCs (DPSCs-shCtr, DPSCs-shB2M, DPSCs-shCIITA) were selected using puromycin (Gibco™, Thermo Fisher Scientific) after 72 h. The knockdown efficiency was confirmed by Western blotting, Immunofluorescence, and flow cytometry, respectively.

### Cell proliferation assay

The self-renewal capacity of DPSCs, DPSCs-shCtr, DPSCs-shB2M, and DPSCs-shCIITA was assessed by Cell Counting Kit-8 (CCK8, Dojindo, Kumamoto, Kyushu, Japan) following the manufacturer's instructions. Briefly, cells were seeded into a 96-well plate at a density of 7000 cells per well and incubated for 1, 3, 5, and 7 days at 37 °C with α-MEM/10% FBS refreshed every two days. On the assessment day, 10% CCK-8 solution was added and incubated for 3 h. Absorbance was measured at 450 nm using a spectrophotometer (SpectraMax M2, Molecular Devices, Sunnyvale, CA, USA).

### Osteogenic differentiation

DPSCs, DPSCs-shCtr, DPSCs-shB2M, and DPSCs-shCIITA were seeded into a 12-well plate at 1 × 10^5^ cells/well. To induce osteogenic differentiation, an osteogenic induction medium consisted of 10% FBS (Thermo Fisher Scientific), 10 Mm—glycerolphosphate (Sigma-Aldrich, Missouri, USA), 10 nM dexamethasone (Sigma-Aldrich), and 50 mg/L L-ascorbic acid (Sigma-Aldrich) was used to induce osteogenic differentiation. The medium was refreshed every 3 days. Cells were then fixed with 4% paraformaldehyde (PFA) for 30 min after a 21-day culture, and 2% Alizarin red (Sigma-Aldrich) was used for 10 min staining. The cells were photographed using a microscope (Nikon, Tokyo, Japan). The Alizarin Red staining area percentage was calculated using the Image J software (Bethesda, Maryland, USA).

### Adipogenic differentiation

DPSCs, DPSCs-shCtr, DPSCs-shB2M, and DPSCs-shCIITA were seeded in a 12-well plate at 1 × 10^5^ cells/well density. Then, the cells were cultured in the adipogenic induction medium (Cyagen Biosciences, Guangzhou, China) for 3 days followed by a 1-day adipogenic maintenance medium (Cyagen Biosciences). After a 21-day culture, cells were fixed with 4% PFA for 30 min and stained with Oil Red O solution (Sigma-Aldrich) for 30 min to identify lipid vacuoles. The Image J software (Bethesda) was applied to quantify the lipid droplet area.

### Neurogenic differentiation

DPSCs, DPSCs-shCtr, DPSCs-shB2M, and DPSCs-shCIITA were seeded into a 12-well plate at a density of 1 × 10^5^ cells/well and cultivated for 7 days in a neurogenic induction medium. The medium contained the components as follows: DMEM/F12: neurobasal [1:1] supplemented with 0.5% [v/v] N2, 1% [v/v] B27, 100 μM cyclic adenosine monophosphate (cAMP), 20 ng/mL basic fibroblast growth factor (bFGF), and 1% penicillin/streptomycin. Tuj1 was served as a neuron marker (#ab78078, Abcam, Cambridge, UK) [28]. All the chemicals were purchased from Thermo Fisher Scientific. The medium was refreshed every 3 days. The immunofluorescence analysis was done using the immunofluorescence method described below. The mean fluorescence intensity of Tuj1 after neurogenic differentiation was measured by Image J software (Bethesda).

### Western blotting

Western blotting analysis for protein expression was performed as previously described [29]. Briefly, the protein extracts were loaded in equal amounts, separated by SDS-PAGE, and then transferred to PVDF membranes. The PVDF membranes were then blocked in 5% milk at room temperature for 1 h and incubated with the primary antibodies listed below (diluted according to manufacturers’ instructions) overnight at 4 °C: anti-B2M (#ab75853, Abcam), anti-CIITA (#sc-13556, Santa Cruz Biotechnology), anti-HLA-ABC (#ab225636, Abcam), anti-HLA-DR (#sc-53319, Santa Cruz Biotechnology), anti-β-actin (#sc-47778, Santa Cruz Biotechnology), and anti-GAPDH (#2118S, Cell Signaling Technology, MA, USA). Following three washes, the secondary antibody conjugated with HRP was used for membrane incubation and then detected by WesternBright ECL HRP substrate (Advansta, San Jose, CA, USA). Image J software (Bethesda) was used for quantification.

### Immunofluorescence

Cells were fixed with either 4% (w/v) cold PFA for 15 min followed by 0.3% (v/v) Triton X-100 in PBS containing 5% FBS permeabilization for 1 h or 100% cold methanol for 5 min followed by PBS containing 5% FBS blocking for 1 h. Cells were then incubated with primary antibodies against HLA-ABC (#ab225636, Abcam), HLA-DR (#sc-53319, Santa Cruz Biotechnology), or Tuj1 overnight at 4 °C. The secondary antibodies employed were Alexa Fluor 488®-conjugated goat anti-mouse antibody and Alexa Fluor 647®-conjugated goat anti-rabbit antibody (Cell Signaling Technology). The nuclei were stained with DAPI (Thermo Fisher Scientific). Images were taken using a fluorescent microscope (Nikon) and quantified by Image J software (Bethesda).

### Flow cytometry

DPSCs-shCtr, DPSCs-shB2M, and DPSCs-shCIITA were collected after treatment and resuspended to approximately 1 mL iced flow cytometry buffer containing 1% PBS, 10% FBS (Thermo Fisher Scientific), and 0.1% sodium azide (S2002-100G, Sigma-Aldrich). Cells were then labelled with primary antibody for 30 min at room temperature in the dark. The anti-HLA-ABC (#ab225636, Abcam) and anti-HLA-DR (#sc-53319, Santa Cruz Biotechnology) were diluted according to the manufacturer's instructions. To remove unbound antibodies, cells were washed with PBS by centrifugation at 1000 rpm for 5 min. Alexa Fluor 488®-conjugated goat anti-mouse antibody and Alexa Fluor 647®-conjugated goat anti-rabbit antibody (Thermo Fisher Scientific) were then added to the cell suspension at the optimal dilution and allowed to incubate for at least 20–30 min at room temperature. The stained cells were analysed by The NovoCyte Advanteon BVR analyser (Agilent Technologies, Santa Clara, California, USA) and FlowJo Software (TreeStar, Ashland, OR, USA).

### Mixed leukocyte-mediated cytotoxicity

To investigate the immunogenicity of DPSCs-shCtr, DPSCs-shB2M, and DPSCs-shCIITA, the leukocyte-mediated cytotoxicity was evaluated after the DPSC and PBMC coculture. Fresh PBMCs were separated from healthy volunteers using density gradient centrifugation with Ficoll-Hypaque (Invitrogen, Thermo Fisher Scientific), the Informed Consents were obtained from all the volunteers before the blood drawing. PBMCs were then resuspended in a culture medium (RPMI 1640, Thermo Fisher Scientific) containing 10% FBS, 50 μmol/l β-mercaptoethanol, nonessential amino acids, and l-glutamine. The 3-day IFN-γ pre-primed DPSCs-shCtr, DPSCs-shB2M, and DPSCs-shCIITA were seeded in a 96-well plate at a density of 1 × 10^4^ cells/well. On the day of the experiment, allogeneic PBMCs were cocultured with DPSCs at a ratio of 12.5:1 in RPMI 1640/10% FBS for 3 days. DPSCs and PBMCs cultured alone were set as the control groups. After coculture, the leukocyte-containing culture medium was collected and centrifuged at 400 × g for 15 min. According to the manufacturer's instructions, the supernatants were incubated with the reaction mixture from the CyQUANTTM LDH Cytotoxicity Assay Kit (ThermoFisher Scientific). The lactate dehydrogenase (LDH) released from damaged cells was proportional to optical density measured at 490 nm with a reference filter of 680 nm. To rule out biased results due to LDH released from damaged PBMCs during coculture, CCK-8 was applied to evaluate the survival of adherent gene-modified cells after removing suspended cells.

### T lymphocyte proliferation assay

Human T cells were negatively isolated from PBMCs using the Pan T cell kit (Miltenyi Biotech, Bergisch Gladbach, Germany) per the manufacturer's instructions. The isolated T cells were assigned to the following experiments:

To evaluate the proliferation of Pan T cells following coculture, isolated T cells were stained with prewarmed 10 μmol/L Vybrant CFDA SE (CFSE)/phosphate-buffered saline staining solution (Invitrogen, Thermo Fisher Scientific) according to the manufacturer's instructions. T cell activation was achieved by mixing the CFSE labelling T cells with ant-CD3/anti-CD28 microbeads (Dynabeads™ Human T-Activator CD3/CD28, Thermo Fisher Scientific) in a 1:1 ratio in RPMI 1640/10% FBS medium. The activated T cells were then cocultured with 3-day IFN-γ pre-primed DPSCs-shCtr, DPSCs-shB2M and DPSCs-shCIITA in the RPMI 1640 medium supplement with 10% FBS, 50 μmol/L β-mercaptoethanol, nonessential amino acids, l-glutamine, and interleukin-2 (IL-2, 30 U/mL, BD Biosciences, San Diego, CA, USA) and incubated in a humidified incubator at 37 °C. After a 4-day co-culture, the T cells were collected for flow cytometry analysis after removing all the microbeads. The control groups were crucial in this experiment: gene-modified DPSCs only, activated CFSE-labelled T cells only, and T cells cultured in the absence of CFSE staining and CD3/CD28 activation. The activated CFDA-labelled T lymphocytes were monitored by flow cytometry after the coculture. The non-proliferating population was determined based on the negative control peak (T cells cultured alone). The quantification of T cell proliferation was reported as proliferating T cell percentage and proliferation index (PI). The PI is calculated by dividing the total number of cell divisions by the number of cells that underwent division. This index specifically accounts for responding cells, meaning it only includes cells that have undergone at least one division. It would be prudent to evaluate this value as it exclusively considers the percentage of cells that have been responsive.

To investigate the CD4 and CD8 expression in Pan T cells after co-culture, isolated T cells were activated by anti-CD3/anti-CD28 microbeads before being co-cultured with gene-modified DPSCs in an incubator for 4 days. After collecting the T cells, the Fc receptor antibody (BD Biosciences) was added to block the T cells for 15 min before a 30-min primary antibody incubation. The primary antibodies were diluted according to the manufacturer's instructions: anti-CD4 (#ab213215, Abcam) and anti-CD8 (#ab17147, Abcam). The secondary antibodies were applied after PBS washing. Flow cytometry analysis was used for CD4 and CD8 expression quantification.

### Statistical analysis

All experiments were completed in triplicates independently, and all data were presented as mean ± standard deviations (SD). Statistical analysis was performed using the GraphPad Prism software version 6.0 (IBM, Armonk, NY, USA). One-way ANOVA with Dunnett's post hoc test or Two-way ANOVA with Bonferroni's post hoc test were used in multiple comparisons. A *p* value ≤ 0.05 was considered statistically significant.

## Results

### Pro-inflammatory conditions upregulate B2M, CIITA, and HLA expression

**Fig. 1:**
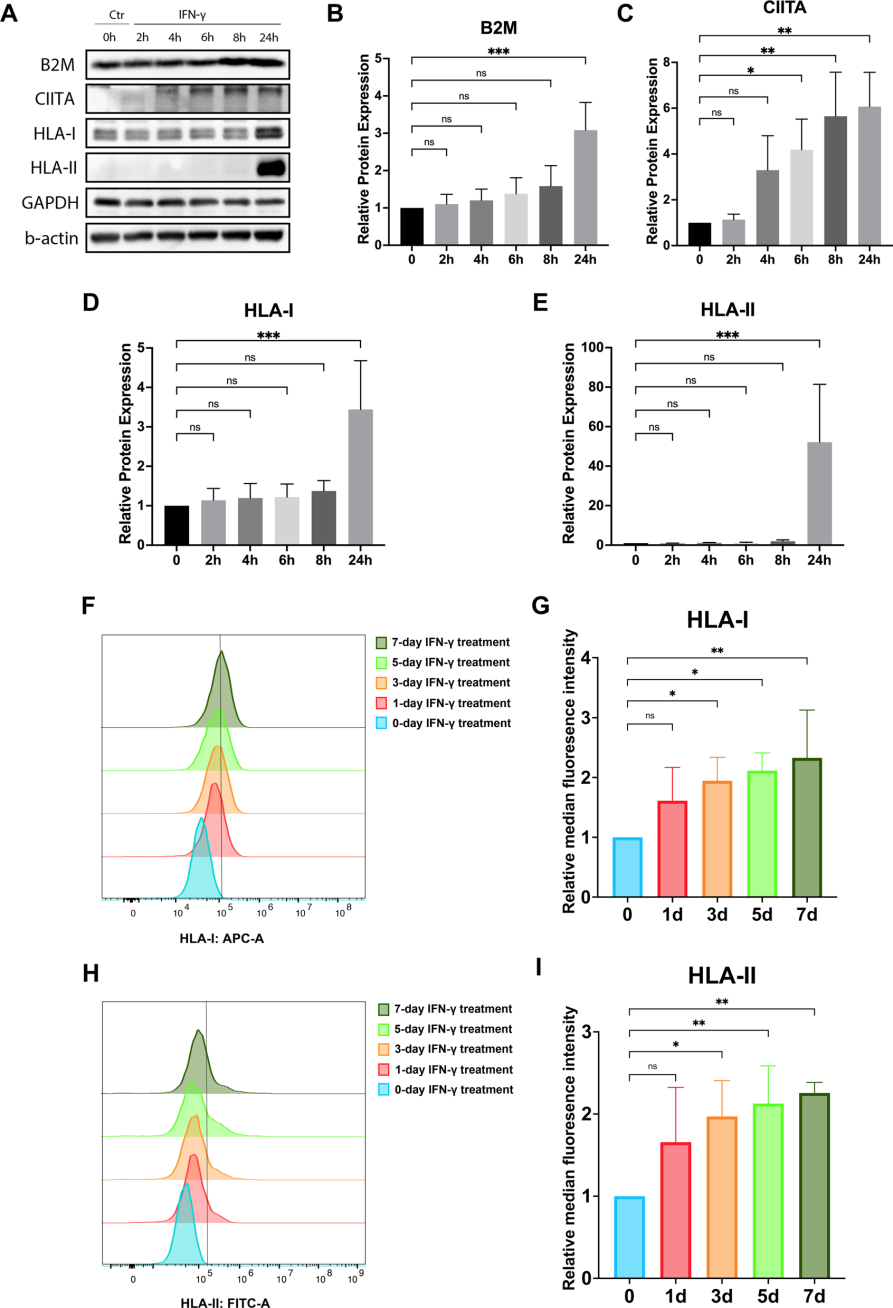
Time-dependent expression of B2M, CIITA, HLA-I, and HLA-II in DPSCs after IFN-γ treatment. **A**–**e** Western blot showed a constant increase in the protein expression of B2M, CIITA, HLA-I, and HLA-II after IFN-γ treatment (10 ng/mL). Images are cropped for visual ease; full-length blots are presented in Additional file 1: Figure S1. **f**–**i** Flow cytometry analysis displayed that the median fluorescence intensity of HLA-I and HLA-II in DPSCs were significantly higher after exposure to IFN-γ compared to the control. Data are represented as mean ± SD (n = 3). ns = no significant difference. *p < 0.05, **p < 0.01, ***p < 0.001

Ten ng/mL of IFN-γ was used in this study to simulate the ischemic inflammatory microenvironment [30]. The concentration was selected according to references and our dose-dependent pilot study [31, 32, Additional file 3: Figure S3]. The western blot results demonstrated a time-dependent, consistent increase in B2M, CIITA, HLA-I, and HLA-II following IFN-γ treatment (Fig. 1a). Compared to the control group, the upregulation of protein levels in B2M and HLA-I was significant only after 24 h IFN-γ treatment (*p* < 0.05, Fig. 1b, d). Meanwhile, the protein expression of CIITA was detectable at 4-h mark following IFN-γ stimulation and significantly increased compared to the control groups at 6 h post-treatment (*p* < 0.05, Fig. 1c). Subsequently, with the elevation of CIITA protein expression, the HLA-II protein level became notably higher than that of the control group after 24-h treatment (*p* < 0.001, Fig. 1e).

The flow cytometry assay confirmed the enhanced expression of HLAs on cell surfaces. The median fluorescence intensity of HLA-I on DPSCs was obtained to be 1.95 ± 0.40 times significantly higher than the control group after exposure to IFN-γ for 3 days and further rose to 2.33 ± 0.80 times that of the control group after 7 days of treatment (*p* < 0.05, Fig. 1f, g). Regarding HLA-II molecules, significant differences were observed after 3 days of IFN-γ stimulation (*p* < 0.05, Fig. 1h, i), with HLA-II median fluorescence intensity reaching 1.97 ± 0.44 times that of the control group. Following a 7-day IFN-γ exposure, it further increased to 2.26 ± 0.13 times the control group’s intensity (*p* < 0.01, Fig. 1h, i).

**Fig. 2:**
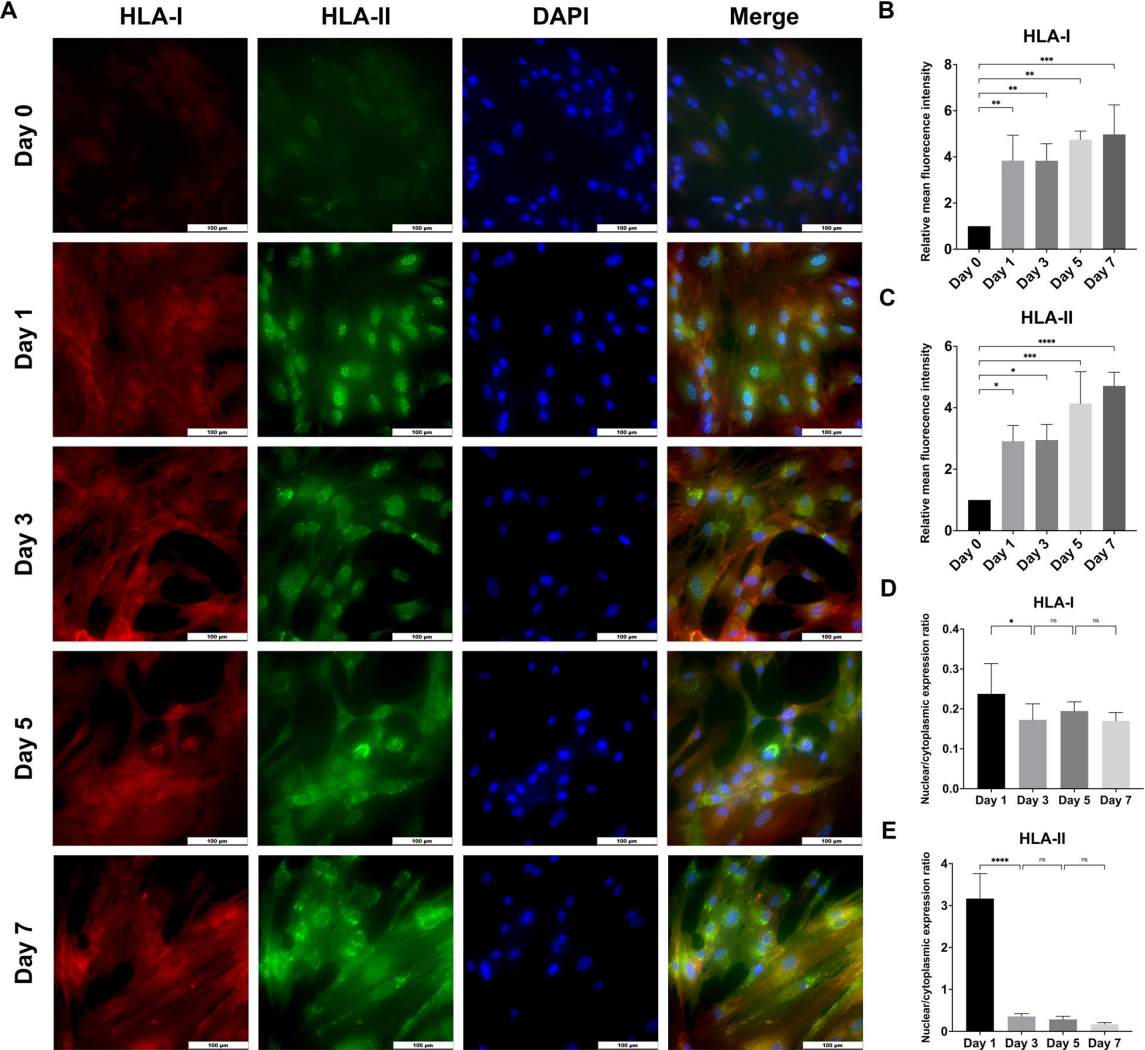
Time-dependent localization of HLA-I and HLA-II in DPSCs after IFN-γ treatment. The cells were fixed using cold methanol. **a** Immunofluorescence images (× 40) of HLA-I and HLA-II at different time points. **b**, **c** Quantification of the mean fluorescence intensity of HLA-I and HLA-II at different time points relative to the intensity observed on Day 0. **d**, **e** The nuclear/cytoplasmic expression ratio of HLA-I and HLA-II was calculated through dividing the fluorescence intensity of HLA molecules in the nucleus by that in the cytoplasm. A ratio greater than 1 indicated the predominant detection of HLA in the nucleus, while a ratio less than 1 suggested an increased accumulation of HLA in the cytoplasm. ns = no significant difference. *p < 0.05, **p < 0.01, ***p < 0.001, ****p < 0.0001

To further investigate the secretory pathway of HLA molecules after IFN-γ treatment, a time-dependent immunofluorescence study was conducted (Fig. 2a). The mean fluorescence intensity of both HLA-I and HLA-II significantly increased after one-day IFN-γ treatment and remained stable over the subsequent 6 days (*p* < 0.05, Fig. 2b, c). A significant increase in the cytoplasmic fluorescence intensity of HLA-I was detected after 3-day IFN-γ treatment (*p* < 0.05, Fig. 2d). However, there was minimal change in the location of HLA-I during the subsequent 4-day period (*p* > 0.05, Fig. 2d). In contrast, the fluorescence of HLA-II was initially detected in the nucleus and gradually diffused to the cytoplasm over time. Following a 3-day IFN-γ treatment, the cytoplasmic accumulation of HLA-II significantly increased when compared to the fluorescence intensity in nuclear (*p* < 0.0001, Fig. 2e). By the 7-day post-stimulation, HLA-II was predominantly located in the cytoplasm with a nuclear/cytoplasmic expression ratio of 0.17 ± 0.04 and exhibited an enhanced fluorescence intensity compared to preceding days.

### Silencing B2M or CIITA downregulate HLA expressions in DPSCs

**Fig. 3:**
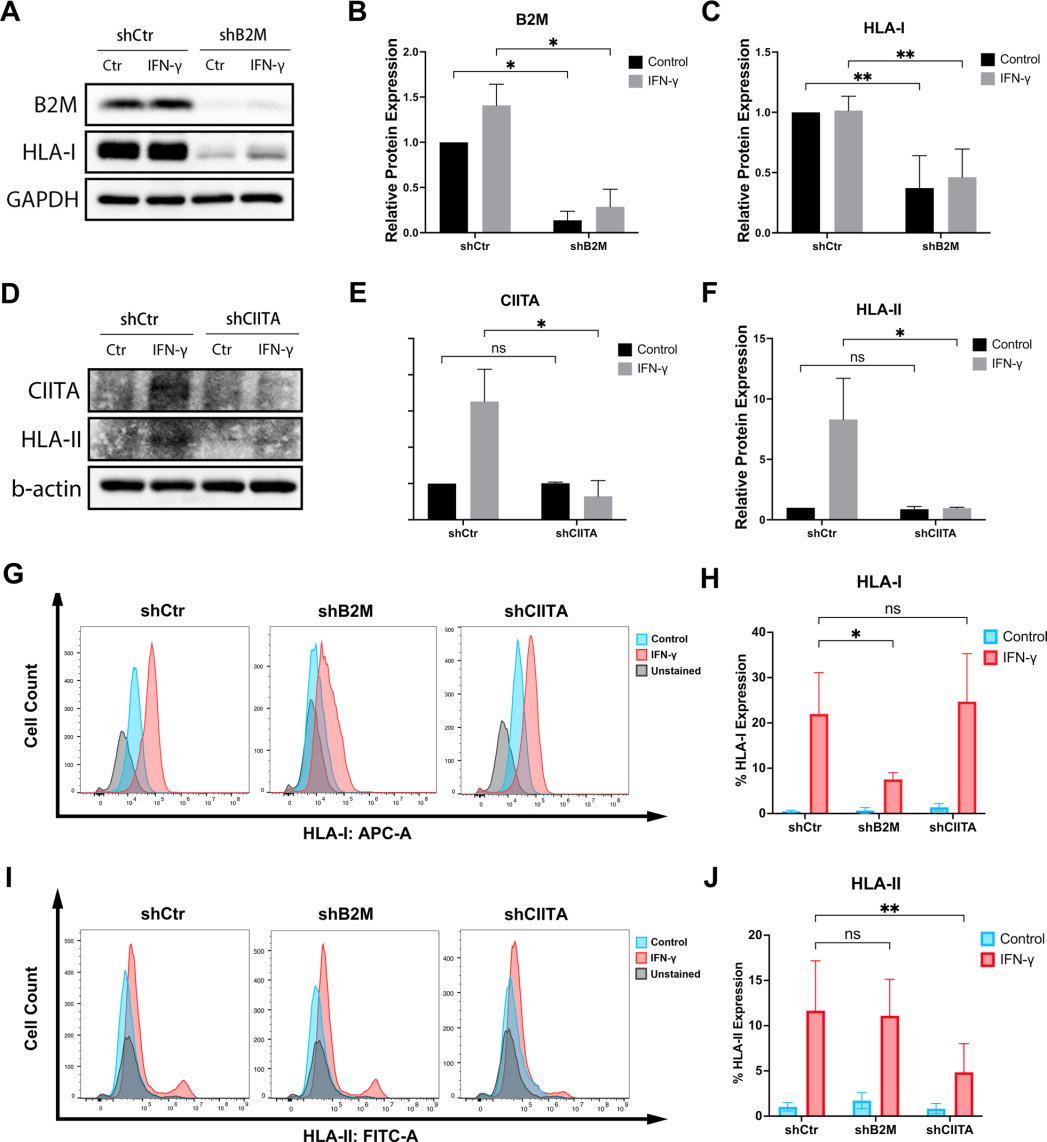
Generation of the gene knockdown DPSCs, cells were exposed to 10 ng/mL of IFN-γ for 24 h for Western blot and immunofluorescence assessment. Flow cytometry was carried out after 72 h of treatment. **a**–**c** Western blot showed reduced protein levels of B2M and HLA-I in DPSCs-shB2M. Images are cropped for visual ease; full-length blots are presented in Additional file 2: Figure S2. **d**–**f** The protein levels of CIITA and HLA-II decreased after CIITA knockdown. **g**, **h** The percentage of positive cells expressing HLA on the DPSC cell surface was evaluated by flow cytometry. Data are represented as mean ± SD (n = 3). *ns* = no significant difference *p < 0.05, **p < 0.01

To verify the role of B2M and CIITA in controlling HLA-I- or HLA-II-mediated responses in DPSCs, B2M or CIITA was knocked down using shRNA particles. Figure 3a, b showed a subtle increase in B2M protein levels in DPSCs-shCtr and DPSCs-shB2M following IFN-γ treatment, with no statistically significant differences (*p* > 0.5). However, a remarkable reduction in B2M expression was observed when comparing DPSCs-shCtr and DPSCs-shB2M groups with or without IFN- γ treatment (*p* < 0.05, Fig. 3a, b). Notably, the inhibition of B2M transcription led to reduced HLA-I protein expression. While HLA-I protein expression showed a slight increase after IFN-γ treatment in both DPSCs-shCtr and DPSCs-shB2M groups (*p* > 0.5), a significant decrease was only evident in the DPSCs-shB2M group, both with and without IFN-γ treatment (*p* < 0.005, Fig. 3c). In contrast, CIITA and HLA-II expression significantly increased in the DPSCs-shCtr group post-IFN-γ treatment. Nonetheless, CIITA knockdown resulted in a considerable suppression of CIITA and HLA-II expression compared to the DPSCs-shCtr group, irrespective of IFN-γ treatment. Compared to the time-dependent experiments shown in Fig. 1, the expression of B2M, CIITA, and HLA-II corresponded with that observed 24 h post-treatment. However, HLA-I exhibited higher expression even without IFN-γ treatment in the DPSCs-shCtr group. This could be attributed to the semi-quantitative nature of Western blots, making them more suitable for demonstrating relative protein expression rather than exact quantities. Consequently, when HLA-I expression in the knockdown group was extremely low, HLA-I expression in the DPSCs-shCtr group appeared relatively high even in the absence of IFN-γ treatment.

Flow cytometry showed that genetically engineered DPSC maintained the downregulation of HLA expression after exposure to IFN-γ for 3 days. The surface expression of HLA-I increased in the DPSCs-shCtr and DPSCs-shCIITA groups. However, it decreased by 14.5 ± 3.7% of cell count following the silencing of B2M (*p* < 0.05, Fig. 3g, h). Similarly, HLA-II expression increased in the DPSCs-shCtr and DPSCs-shB2M groups but decreased significantly to 4.9 ± 2.9% (*p* < 0.005) of cell count after silencing of CIITA compared to DPSCs-shCtr (Fig. 3i, j).

**Fig. 4:**
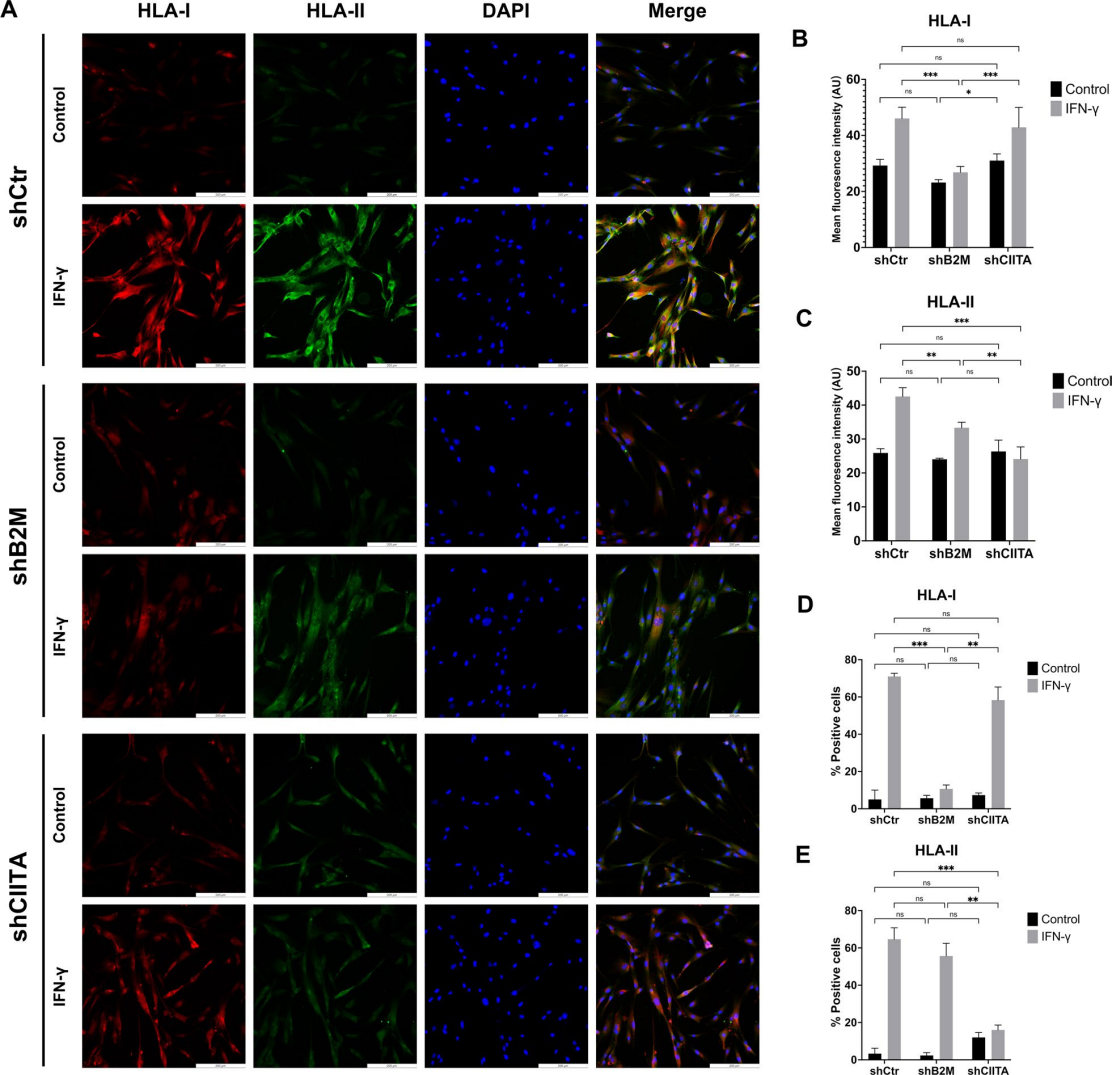
Immunofluorescent detection of HLA-I and HLA-II expression in gene knockdown DPSCs. The cells were fixed using 4% PFA. **a** Immunofluorescence images (× 20) of HLA-I and HLA-II expression in DPSCs with genetic modification. The HLA-I and HLA-II were stained with red and green fluorescence, respectively. The scale bar represents 200 μm. **b**, **c** Quantification of HLA-I and HLA-II mean fluorescence intensity in genetically modified DPSCs with or without the IFN-γ treatment. **d**, **e** Quantification of HLA-I and HLA-II positive cell numbers in genetically modified DPSCs with or without the IFN-γ treatment*. ns* = no significant difference. *p < 0.05, **p < 0.01, ***p < 0.001

In the presence of IFN-γ treatment, both the fluorescence intensity and the percentage of positive cells expressing HLA-I were significantly reduced in B2M knockdown groups compared to the other two groups (*p* < 0.01, Fig. 4a, b, d). A significant reduction in HLA-I fluorescent intensity was only found in DPSCs-shB2M group compared to the DPSCs-shCIITA group when IFN-γ treatment was not applied (*p* < 0.05, Fig. 4a, b, d). Similarly, the fluorescence intensity and the percentage of positive cells expressing HLA-II decreased significantly following IFN-γ treatment in the CIITA knockdown groups compared to others (*p* < 0.01, Fig. 4a, c, e). However, the DPSCs-shB2M group also showed a significant reduction in HLA-II fluorescent intensity compared to the control groups, although it remained significantly higher than the DPSCs-shCIITA groups (*p* < 0.01, Fig. 4a, c, e).

### The self-renewal capacity of DPSCs was not compromised after B2M or CIITA silencing

**Fig. 5:**
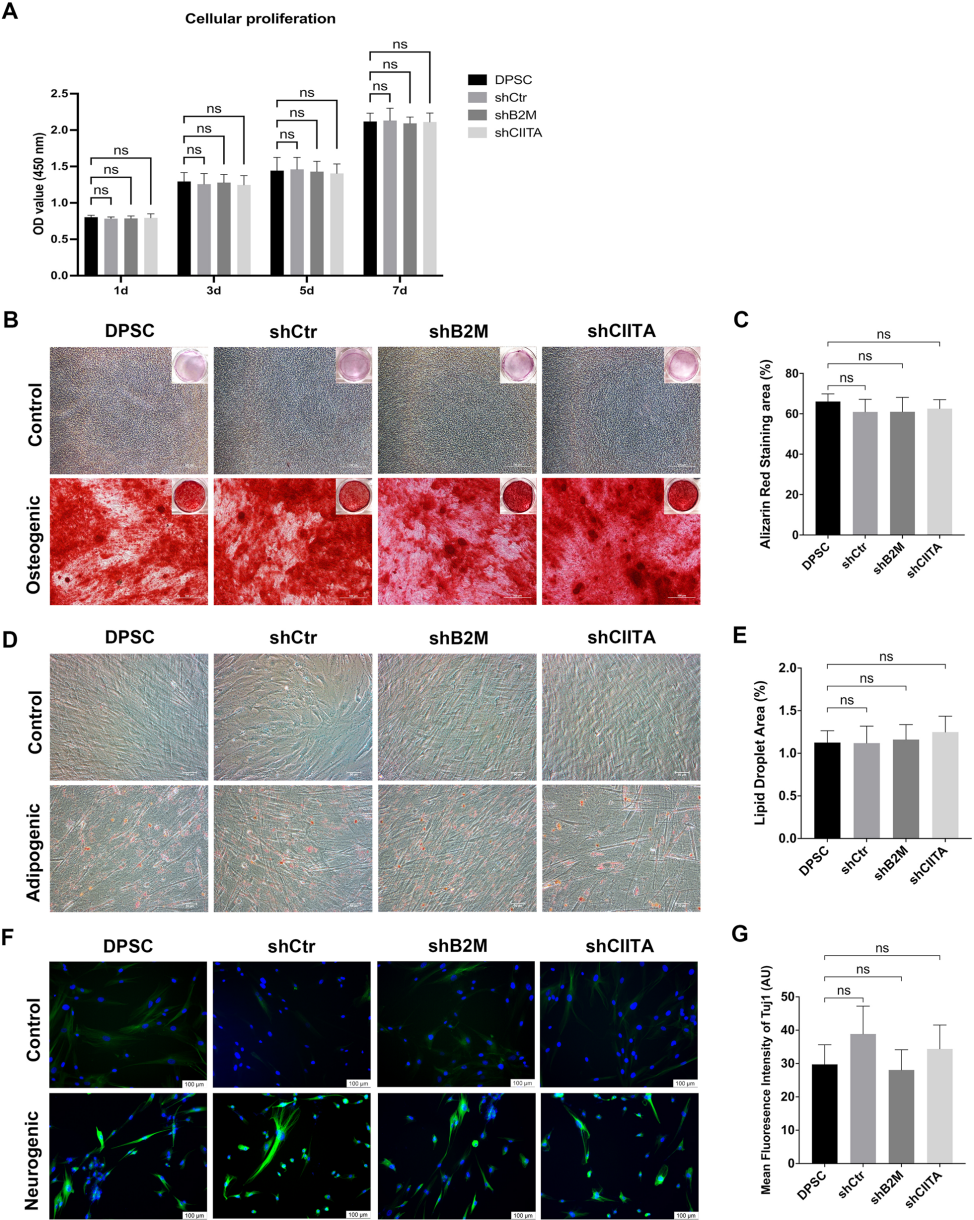
Characterization of genetically modified DPSCs. **a** CCK8 assay analysis of the proliferation of DPSCs, DPSCs-shCtr, DPSCs-shB2M, and DPSCs-shCIITA at different time points. **b, c** The osteogenic differentiation assay (× 4) stained calcium deposits with Alizarin Red. Image J software was used to quantify the percentage of Alizarin Red staining area. **d**, **e** Adipogenic differentiation assay (× 20), the lipid droplets were stained with Oil red O, and the lipid droplet area percentage was calculated by Image J software. **f**, **g** Neurogenic differentiation assay (× 20), the green fluorescence denoted Tuj1, and the blue fluorescence denoted DAPI. The mean fluorescent intensity of Tuj1 in different groups was computed by utilizing Image J software. *ns* = no significant difference. Data are represented as mean ± SD (n = 3)

The self-renewal capacity of DPSCs-shB2M and DPSCs-shCIITA were evaluated by CCK8 proliferation assay. The growth rate and dynamics of lentiviral particle treated DPSCs were found to be comparable to those of the parental cell line (*p* > 0.05, Fig. 5a). Transduction with lentiviral particles did not result in any significant alteration in DPSCs’ self-renewal ability.

### The pluripotency of DPSCs was maintained after silencing B2M or CIITA

Following 3-week osteogenic induction, mineralized matrix formation was observed in DPSCs, regardless of lentiviral particle transductions. Calcium nodules were stained by Alizarin Red staining in the osteogenic groups (Fig. 5b). In terms of adipogenic differentiation, numerous lipid droplets were stained with Oil Red O in all groups, except for the control group cultured without adipogenic induction medium (Fig. 5d). Neurogenic differentiation was also confirmed in all groups after 7 days of treatment by immunofluorescence (Fig. 5f). No significant differences in differentiation capacity were detected among different cell lines (Fig. 5c, e, g).

These results collectively indicated that DPSCs with genetic modification retained the capacity to differentiate into osteoblasts, adipocytes, and neurocytes.

### Leukocyte-mediated cytotoxicity was reduced after B2M or CIITA was knockdown in DPSCs

**Fig. 6:**
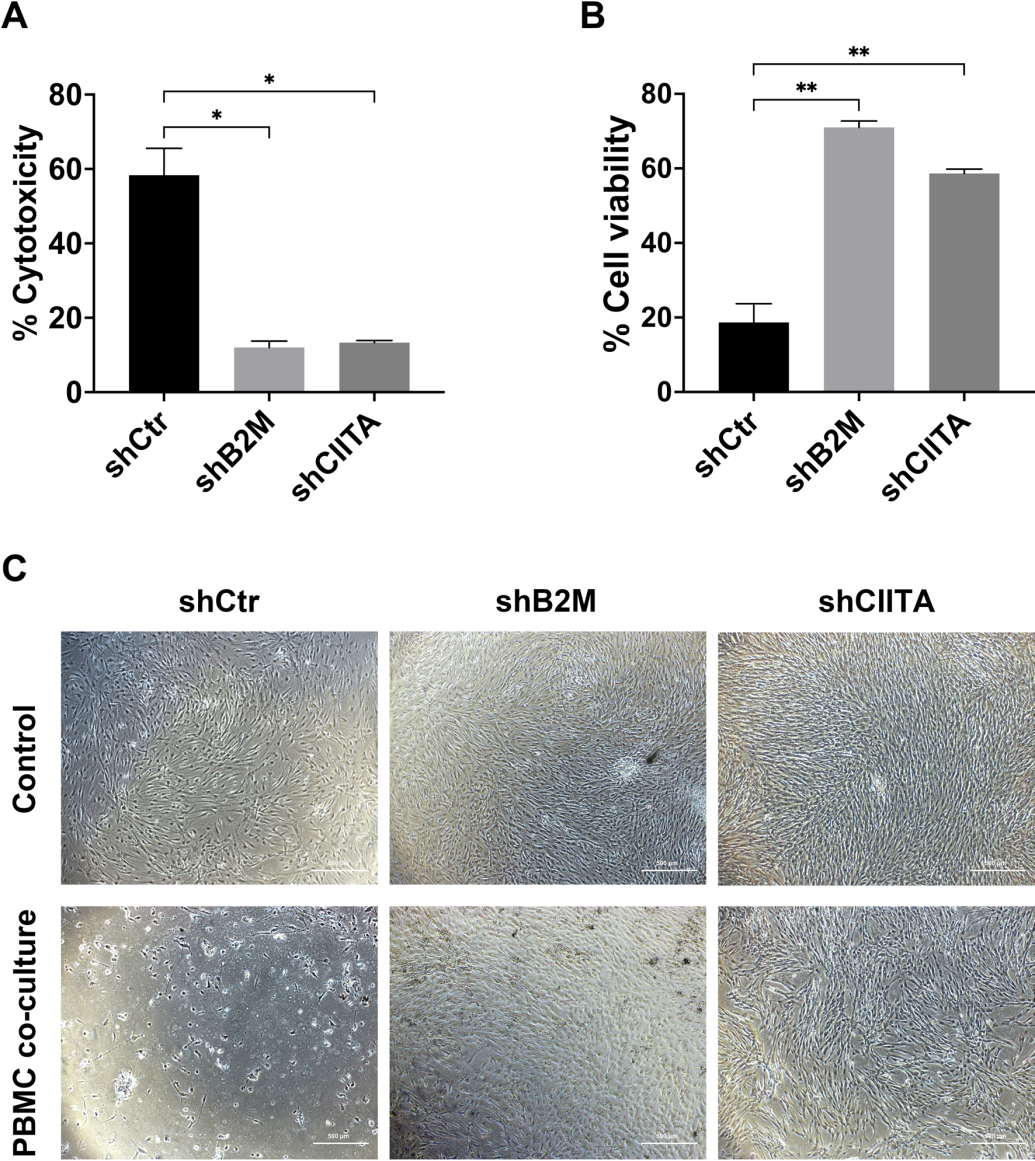
Quantification of the cytotoxicity and the viability in different types of cells after co-culture with PBMCs. **a** After 3-day co-culture, the cytotoxicity (LDH release) was more significant in DPSCs-shCtr groups than in DPSCs-shB2M and DPSCs-shCIITA groups. **b** PBMCs were removed and the viability of surviving cells was evaluated by CCK8 assay. **c** The microscopic images (× 4) of surviving DPSCs after co-culture. Data are represented as mean ± SD (n = 3). *p < 0.05, **p < 0.01

After 3-day coculture, the leukocyte-mediated cytotoxicity was significantly higher in the DPSCs-shCtr groups (58.3 ± 7.2%) compared to the DPSCs-shB2M and DPSCs-shCIITA groups (12.0 ± 1.7% and 13.3 ± 0.6% respectively) (*p* < 0.05, Fig. 6a). Upon removal of all suspended cells, a greater percentage of DPSCs-shB2M (71.0 ± 1.7%) and DPSCs-shCIITA (58.7 ± 1.2%) survived in the coculture with PBMC, whereas over 80% of DPSCs-shCtr were found to be dead (*p* < 0.01, Fig. 6b).

The survival of DPSCs was also visualized through microscopic images as shown in Fig. 6c. In the control groups without PBMC coculture, a dense population of spindle-shaped cells attaching to the cultured dishes was observed. In contrast, after PBMC coculture, DPSCs-shCtr displayed significantly lower cell density with various morphological changes, such as cell shrinkage and detachment. Conversely, the DPSCs-shB2M and DPSCs-shCIITA after PBMC coculture remained healthy in appearance with regular shape and size (Fig. 6c).

### Either B2M or CIITA knockdown inhibited the proliferation of allogeneic T lymphocytes

**Fig. 7:**
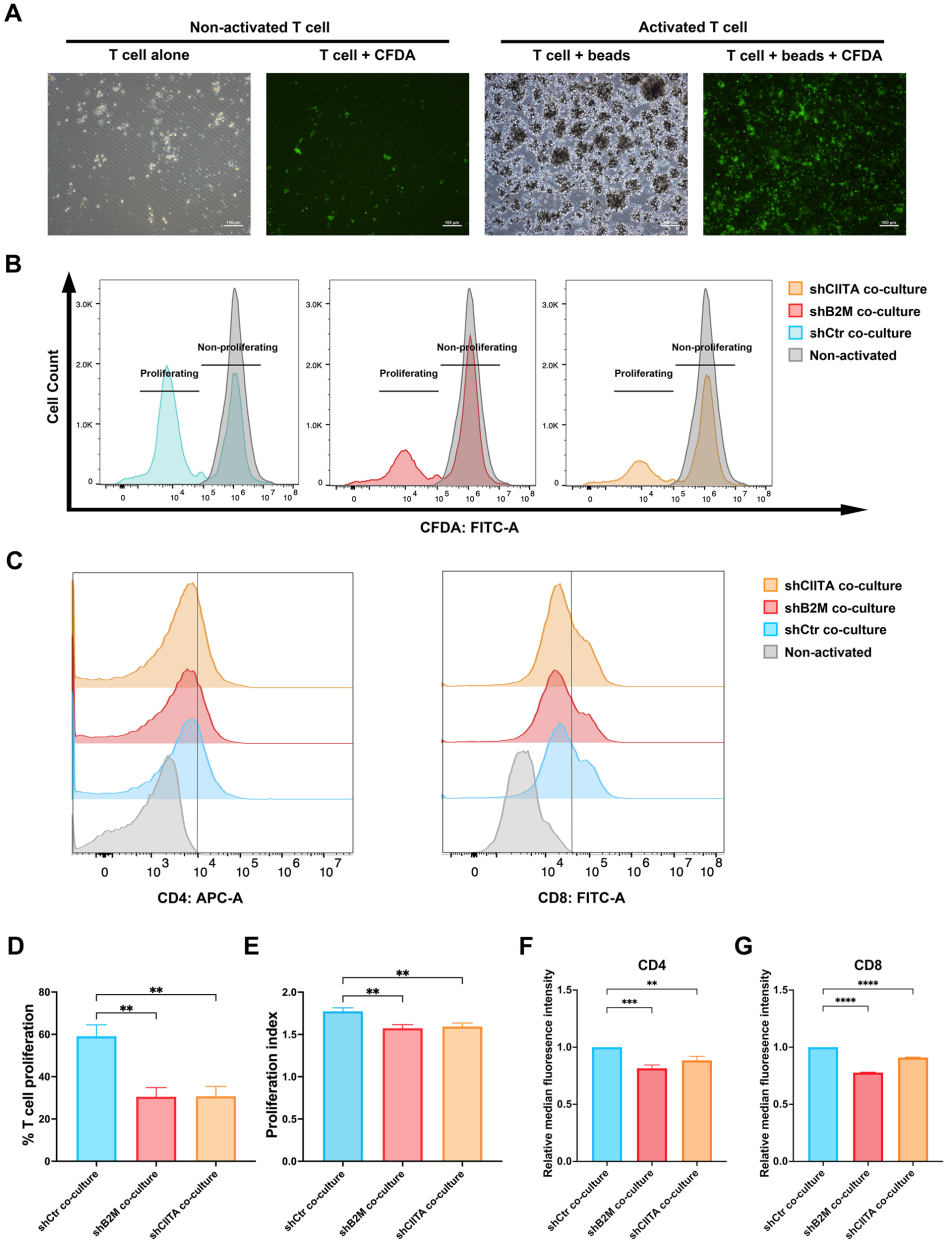
T cell proliferation and CD4 and CD8 expression in the presence of DPSCs-shCtr, DPSCs-shB2M, and DPSCs-shCIITA. **a** The microscopic images of non-activated T cells and activated T cells with or without CFDA fluorescent. **b** CFDA fluorescent peaks of T cells activated in the presence of DPSCs. The grey fluorescent peak represents the parent population. **c** Flow cytometry histograms showed a decrease in CD4 and CD8 T cells in DPSCs-shCIITA or DPSCs-shB2M groups compared to the control group following a 4-day co-culture. **d**, **e** Flow cytometry quantification revealed a higher mean percentage of proliferating T cells and proliferation index (PI) in DPSCs-shCtr co-cultured groups. **f**, **g** Flow cytometry quantification illustrated a higher median fluorescence intensity of CD4 and CD8 T cells in the DPSCs-shCtr group after 4 days of co-incubation. Data are represented as mean ± SD (n = 3). **p < 0.01, ***p < 0.001, ****p < 0.0001

The isolated T cells were activated by anti-CD3 and anti-CD28 beads. The activation was confirmed by their morphology under optical and fluorescent microscopes. A significant cluster of T cells and magnetic beads were viewed after activation compared to the non-activated T cells. The size of activated T cells also increased during proliferation (Fig. 7a).

A lower peak of non-proliferating population was observed with a higher proliferating rate (Fig. 7b). T cells cocultured with DPSCs-shCtr that were exposed to IFN-γ had the highest number of proliferating T lymphocytes (59.1 ± 5.4%) and PI (1.77 ± 0.04), while downregulation of B2M and CIITA in DPSCs significantly inhibited the proliferation of T lymphocytes, in which demonstrated lower proliferating T cell percentage (30.5 ± 4.4%, 30.7 ± 4.7%, *p* < 0.01) and PI (1.57 ± 0.04, 1.59 ± 0.04, *p* < 0.005, Fig. 7d, e).

To further explore the consequence of T lymphocyte proliferation after co-culture, the median fluorescence intensities of CD4 and CD8 were quantified by flow cytometry (Fig. 7c). The results showed that the intensity of CD4 significantly diminished in DPSCs wherein B2M and CIITA were knocked down, compared to the control groups (*p* < 0.01, Fig. 7f). Meanwhile, the intensity of CD8 also showed a significant decrease in DPSCs-shB2M and DPSCs-shCIITA co-cultured groups (*p* < 0.0001, Fig. 7g). These results support the hypothesis that B2M or CIITA knockdown can maintain immunoprivilege in DPSCs.

## Discussion

Stem cell therapy is widely acknowledged as an effective regenerative technique for treating pulpitis, apical periodontitis, and other ischemic disease. Previous research has investigated autologous adipose-derived or bone marrow-derived MSCs for pulp regeneration. Nonetheless, the regenerated pulp tissue in these studies exhibited greater mineralization with reduced angiogenesis and innervation compared to the pulp tissue generated by DPSCs [33]. Therefore, DPSCs remain the preferred cell source for pulp regeneration. A recent preclinical study using granulocyte colony-stimulating factor (G-CSF)-mobilized DPSCs (MDPSCs) obtained under good manufacturing practice conditions for autologous transplantation was shown to be safe and effective [34]. However, the limited resources of discarded human teeth and the high cost and time-consuming nature have impeded the progress of autologous MDPSC transplantation. Also, some studies have provided valuable insights into the immunomodulatory properties and regenerative potential of DPSCs, suggesting that allogeneic DPSCs can suppress the alloantigen-induced immune response without triggering an alloimmune response [35–37]. However, these studies did not answer how these cells might respond immunogenically in challenging microenvironments after allotransplantation. Therefore, uncertainties remain regarding the potential rejection of the allogeneic DPSCs due to harsh microenvironments or cell differentiation. Given these concerns, using banked allogeneic DPSCs with B2M and CIITA knockdown would bring significant benefits in terms of time and cost savings and improve the clinical practicality of allogeneic DPSC transplantation.

Various inflammatory cytokines have been observed in inflammatory lesions such as periapical lesions [38]. Even after undergoing disinfection and anti-inflammatory treatment, residual quantities of these cytokines may persist. Additionally, MSCs are promptly exposed to an inflammatory microenvironment upon transplantation. Among these inflammatory cytokines, IFN-γ is special for its ability to induce MHC expression, making it particularly relevant to transplantation [39]. Therefore, we used IFN-γ as an inflammatory stimulus in this study and found that DPSCs constantly increased B2M and HLA-I expressions over time. HLA-I is typically expressed in nucleated cells, and the harsh microenvironment upregulated its expression [22]. HLA-II expression was absent in the untreated DPSCs and peaked after 24 h of exposure to IFN-γ. This corroborated the previous studies that HLA-II was only expressed in MSCs after receiving various stress signals, such as pro-inflammatory stimulation or hypoxia [40, 41]. It was fascinating that the cell surface expression of HLA was only detectable after 1 day treatment and reached its maximal expression after 5 to 7 days assessed by flow cytometry. Theoretically, HLA-I and II synthesis occurs in the endoplasmic reticulum and is transported through the Golgi compartment to the cell surface. Therefore, cell surface expression lagged. This could explain why significant differences in HLA levels were detected after 1 day of IFN-γ treatment when measured by Western blotting but could only be detected after 3 days of IFN-γ treatment by flow cytometry, as Western blotting quantified total HLA protein levels in DPSCs, whereas flow cytometry specifically recognizes HLA proteins expressed on the cell surface. We also observed by immunofluorescent staining that HLA-II was initiated within the nucleus and subsequently migrated to the cytoplasm. The localization of HLA within the nucleus is unexpected, as its typical site of synthesis is within the endoplasmic reticulum. This issue may be explained by a study from Stadler et al., in which they compared the immunofluorescence and fluorescent protein tagging techniques and found that the immunofluorescence was less effective in localizing the proteins to endomembrane structures. Immunofluorescence could wrongly show proteins in the nucleus despite being presented in the endoplasmic reticulum or Golgi apparatus [42]. Therefore, it is reasonable to assume that the HLA-II fluorescence observed in the nucleus originated in the endoplasmic reticulum. Assessing the nuclear/cytoplasmic ratio facilitated the quantitative evaluation of HLA-II translocation from the endoplasmic reticulum to the cell membrane.

In this study, we first reported a proposed approach to reduce DPSC immunogenicity using lentiviral particles to silence B2M and CIITA. Our results revealed that silencing B2M and CIITA inhibited the HLA expression in the pro-inflammatory microenvironment, confirming the critical role of B2M and CIITA in regulating the HLA-I and HLA-II expression. However, the HLA expression level remained slightly elevated in gene knockdown DPSCs. This may be attributed to the RNAi gene knockdown technique, which did not completely eliminate HLA expression. Several studies reported that the increased susceptibility to infections was found to be linked to a deficiency of HLA expression [43, 44]; residual HLA expression may be helpful in preventing NK cell cytotoxicity [45]. Therefore, gene editing technologies like CRISPR/Cas9 that entirely delete the designated gene might not be the optimal choice for our single knockdown study.

Our study indicated that the DPSCs-B2M exhibited a significant decrease in the expression of B2M and HLA-I even in control groups that did not receive IFN-γ treatment. However, the expression of CIITA and HLA-II in control groups remained unchanged after CIITA knockdown. It has been widely reported that the HLA-I is naturally presented on the surface of most nucleated cells [46], and B2M inhibition resulted in reduced expression regardless of the presence of IFN-γ or not. On the contrary, HLA-II expression is typically linked to infection, inflammation, or trauma, which is commonly found in antigen-presenting cells, B lymphocytes, and activated T lymphocytes [47]. These findings suggested that DPSCs could potentially serve as antigen-presenting cells following transplantation. Therefore, knockdown of B2M and CIITA, particularly CIITA, may assist in immunological privilege.

Following genetic modification, HLA-silenced and non-silenced DPSCs, as well as native DPSCs, displayed comparable morphological features and growth dynamics. The pluripotency of cells remained after transduction and knockdown, indicating that the knockdown of B2M and CIITA did not impact the mesenchymal characteristics of DPSCs. This is essential to ensure the application of genetically modified DPSCs in clinical translation. In addition, differentiation would induce the transplanted cells to express HLA-II, thereby compromising the survival of transplanted allogeneic MSCs [21]. The proposed approach using lentiviral vectors to silence B2M and CIITA to reduce the immunogenicity of DPSCs can be easily integrated into the cell expansion process during the production of therapeutic DPSCs, which potentially overcomes the challenges of immune rejection with transplanting allogeneic DPSCs [48].

Our study used the IFN-γ pre-primed DPSCs in co-culture with PBMC or T cells to evaluate the intensity of the alloimmune response. It has been reported that, despite the initial increase in MHC expression following allotransplantation, IFN-γ plays a protective role in maintaining the viability of transplanted cells or tissue by promoting angiogenesis [49]. Previous studies on the impact of priming on allogeneic MSCs have shown that IFN-γ-primed MSCs demonstrate enhanced immunosuppressive abilities, effectively inhibiting T cell function and improving the survival of transplanted cells or tissue [50, 51]. In accordance with these findings, we preactivated the DPSCs with IFN-γ to enhance their survival rate.

After transplantation, leukocytes, specifically T cells, play an integral role in identifying and combating transplanted tissue. Upon recognizing unfamiliar antigens, such as mismatched HLAs, T cells become activated on the surface of the transplanted tissue through their T cell receptors (TCRs). Following activation, T cells undergo clonal expansion and release various effector molecules contributing to immune rejection [52–54]. Cytotoxic CD8 T cells directly eliminate cells that are deemed unfamiliar. They release cytotoxic molecules, including perforin and granzymes, which prompt cell death in the transplanted tissue [55, 56]. On the other hand, helper CD4 T cells provide support and coordination for immune responses. They release cytokines (IL-2), which encourage the activation and growth of other immune cells involved in rejection, including cytotoxic CD8 T cells [57]. Our data has confirmed this process, as the coculture of allogeneic leukocytes with DPSCs-shCtr led to decreased cell viability and increased cytotoxicity. There was a residual cytotoxicity of approximately 10% and a cell death rate ranging from 30 to 40% detected in both DPSCs-shB2M and DPSCs-shCIITA groups. This might be attributed to the activation of NK cells after the reduction in the expression of HLA-I [58]. The remaining function of cytotoxic CD8 T cells may also play a role in the cell death observed in the DPSCs-shCIITA group. Moreover, the investigation into the impact of B2M and CIITA knockdown on activated T cell proliferation indicated that the reduced expression of either B2M or CIITA resulted in lower rates of T cell proliferation. Our study further revealed that CD8 and CD4 T cells were significantly suppressed after being cocultured with DPSCs-shB2M and DPSCs-shCIITA. These results suggested that the downregulation of HLA expression in DPSCs provided a remarkable advantage in circumventing host immune system attacks, thereby enhancing the survival of transplanted cells.

The study also showed a reduction of CD4 and CD8 T lymphocytes in the DPSCs-shB2M and DPSCs-shCIITA groups, where HLA-I and HLA-II expressions were silenced. The intricate interplay between CD4 T cells and CD8 T cells is responsible for the observed phenomenon. CD4 T cells secrete cytokines such as IL-2 for CD8 T cell activation, while CD8 T cells release cytokines such as IFN-γ to activate and differentiate CD4 T cells [59, 60]. The deficiency of MHC-I can potentially affect the activation and proliferation of CD4 T cells by impeding the proliferation of CD8 T cells. The inverse relationship is also genuine. The impaired proliferation of CD4 T cells may directly impact the activation and proliferation of CD8 T cells. Therefore, CD8 T cell function was inhibited even in the DPSCs-shCIITA groups, leading to a comparable cell survival rate as that in the DPSCs-shB2M groups. This could be further verified by detecting the pro-inflammatory cytokines in various genetically modified cells.

This study has some limitations, including the absence of animal studies to verify the rejection of allogeneic DPSCs in the inflammatory microenvironment. In fact, the animal study was postponed because of the difficulty in applying for ethics approval due to COVID-19, but it is currently in progress. Additionally, although some studies suggested that, with the advanced modern technique, gene knockdown can be performed without inducing tumorigenesis with precise targeting and controls [61–63], the potential tumorigenesis remains a recognized limitation. Further thorough investigations in this area, such as specifying the insertion site, should done to ensure the safe translation of our findings into clinical applications. Future works should also focus on generating the B2M and CIITA dual knockdown DPSCs, thereby improving the survival rate of transplanted DPSCs. The presence of minor histocompatibility antigens (mHAgs) responsible for the alloimmune response should also be investigated to minimize the alloimmune response during transplantation. Furthermore, an in vivo study utilizing engineered DPSCs is a viable option for future investigations.

## Conclusions

Genetic knockdown of B2M or CIITA is a viable way to generate low immunogenicity recognizable DPSCs without affecting their stem cell characteristics. This might provide a novel strategy for applying allogenic DPSCs in tissue regeneration.

## Supplementary Information

Additional file1 (JPG 419 KB)

Additional file2 (JPG 326 KB)

Additional file3 (JPG 6713 KB)

## Data Availability

The datasets used and/or analyzed during the current study are available from the corresponding author on reasonable request.

## Abbreviations

B2M: Beta 2-microglobulin
CCK8: Cell Counting Kit-8
CFDA-SE: Carboxyfluorescein diacetate succinimidyl ester
CFSE: Carboxyfluorescein succinimidyl ester
CIITA: Class II histocompatibility complex transactivator
DAPI: 4′,6-Diamidino-2-phenylindole
DPSC: Dental pulp stem cells
FBS: Fetal bovine serum
GAPDH: Glyceraldehyde-3-phosphate dehydrogenase
HLA: Human leukocyte antigen
HRP: Horseradish peroxidase
IFN-γ: Interferon gamma
IFU: Increasing infectious units
IL-2: Interleukin-2
LDH: Lactate dehydrogenase
MHC: Major histocompatibility complex
MSC: Mesenchymal stem cell
Tuj1: Beta tubulin III
PBMC: Peripheral blood mononucleated cell
PBS: Phosphate-buffered saline
PFA: Paraformaldehyde
PI: Proliferation index
PVDF: Polyvinylidene fluoride
SDS-PAGE: Sodium dodecyl-sulfate polyacrylamide gel electrophoresis
