# Supplementary figures and images for: B2M or CIITA knockdown decreased the alloimmune response of dental pulp stem cells: an in vitro study
Source: Stem Cell Res Ther. 2024 Nov 14;15:425. doi: 10.1186/s13287-024-04023-5 (PMC11562604; doi:10.1186/s13287-024-04023-5)

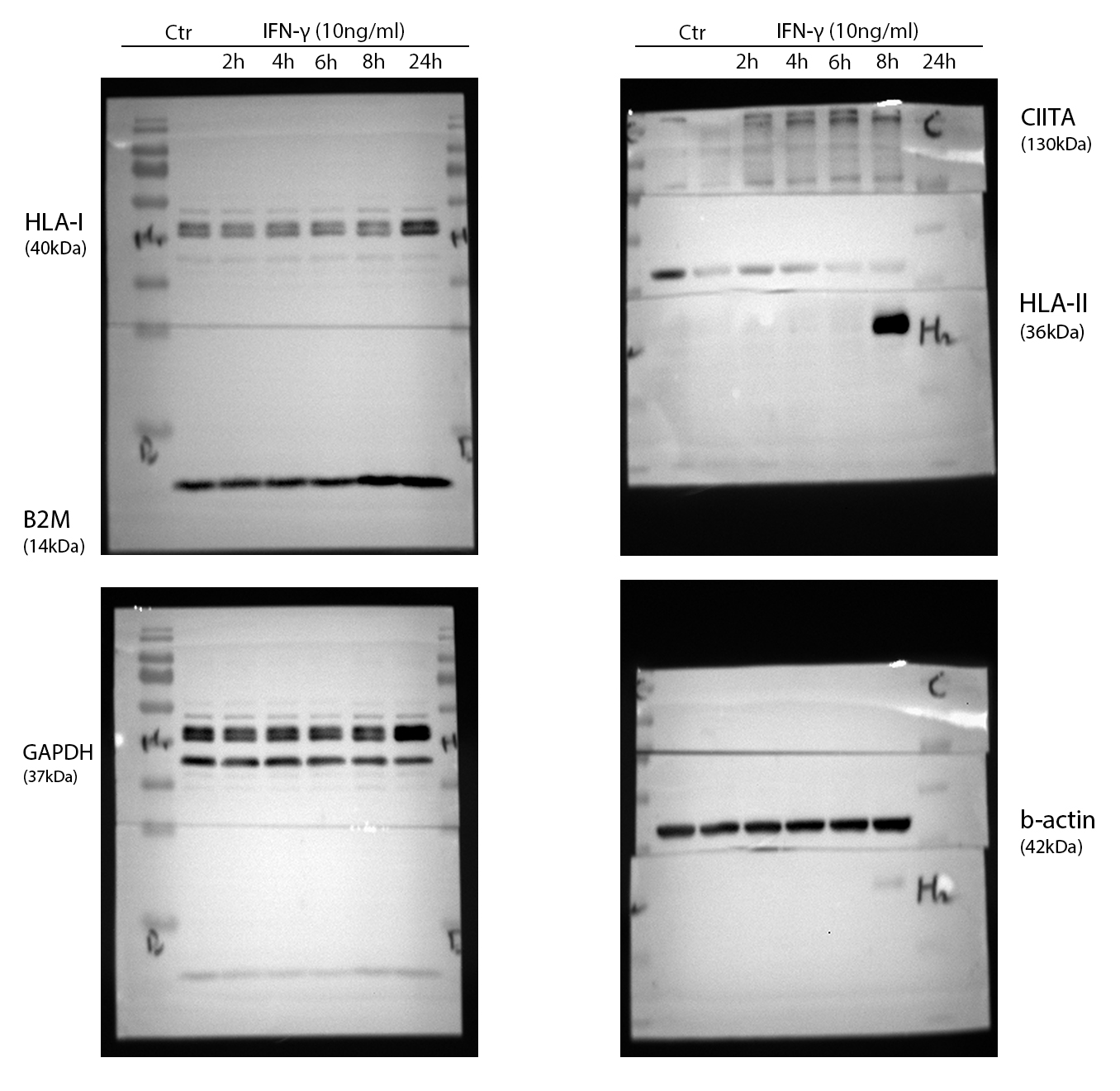

Supplement: Supplementary file 1 — Additional file1 (JPG 419 KB) [file 13287_2024_4023_MOESM1_ESM.jpg]

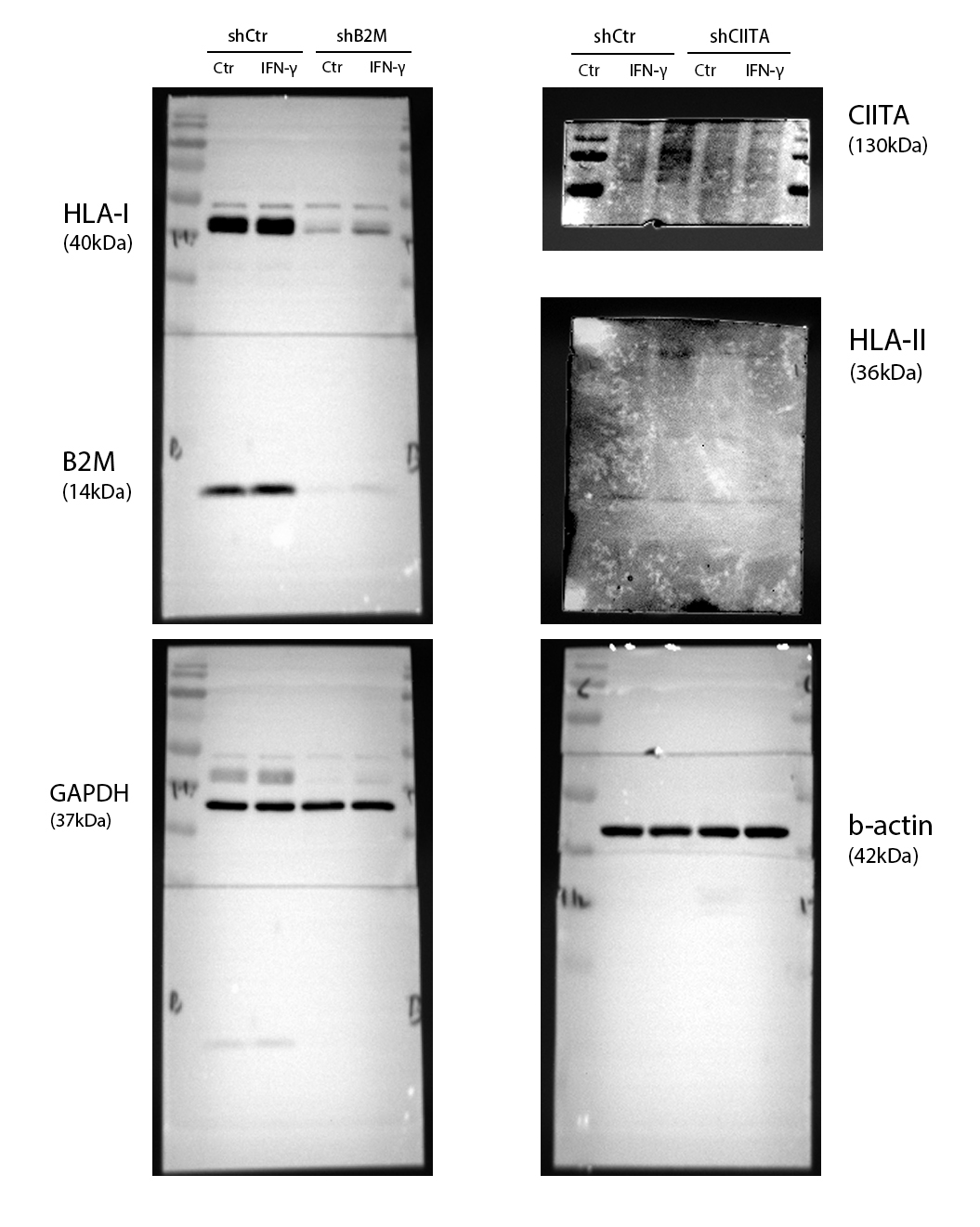

Supplement: Supplementary file 2 — Additional file2 (JPG 326 KB) [file 13287_2024_4023_MOESM2_ESM.jpg]
